# *Tna*o38, high five and *Sf*9—evaluation of host–virus interactions in three different insect cell lines: baculovirus production and recombinant protein expression

**DOI:** 10.1007/s10529-013-1429-6

**Published:** 2013-12-29

**Authors:** Monika Wilde, Miriam Klausberger, Dieter Palmberger, Wolfgang Ernst, Reingard Grabherr

**Affiliations:** Vienna Institute of BioTechnology (VIBT), University of Natural Resources and Life Sciences, Muthgasse 11, 1190 Vienna, Austria

**Keywords:** *Autographa californica* nuclear polyhedrosis virus, Baculovirus expression vector system, Baculovirus titer determination, Hemagglutinin of influenza virus, Host–virus interactions, Influenza viral protein, Insect cell lines

## Abstract

Purpose of work: A comparative analysis of new and established insect cell lines, in regard to process relevant parameters, provide data that can be exploited for designing more robust and effective protein production processes. The baculovirus-insect cell expression system has been efficiently used for the production of heterologous proteins. Three different insect cell lines *Tnao*38, High Five and *Sf*9 were compared in terms of virus susceptibility, baculovirus production and product yield of an intra-cellularly (YFP) and extra-cellularly (influenza A virus hemagglutinin)-expressed recombinant protein. The *Tnao*38 and High Five cell lines exhibited higher (tenfold) susceptibility to baculovirus infection than *Sf*9 cells, whereas *Sf*9 cells showed a higher (100-fold) capacity for production of infectious virus particles. Analysis of recombinant protein expression revealed considerably higher product yields in *Tnao*38 and High Five cells as compared to *Sf*9 cells, for both model proteins. Overall, the two *Trichoplusia ni*-derived cell lines, High Five and *Tnao*38, were significantly more efficient in terms of secreting proteins such as the glycoprotein hemagglutinin of influenza A virus.

## Introduction

The insect cell-based baculovirus expression vector system (BEVS) is a widely used and safe tool for the production of recombinant proteins and baculovirus particles (Kost and Condreay 1999; Miller 1997). Production is fast and easy and provides high yields, efficient protein secretion and eukaryotic-like glycosylation (Kost et al. 2005). Furthermore, BEVS offers the possibility to co-express several proteins, thereby allowing the production of multi-subunit proteins such as transcription factor complexes or virus-like particles (Berger et al. 2004; Bieniossek et al. 2008; Fernandes et al. 2013; Fitzgerald et al. 2006; Kang et al. 2009; Trowitzsch et al. 2010) which makes it a unique system for many applications in basic research, biotechnology and biopharmacy. The most widely used baculovirus, *Autographa californica* nuclear polyhedrosis virus (*Ac*NPV), infects about 40 different Lepidoptera belonging to 11 different families (Doyle et al. 1990). Although, the host range of *Ac*NPV is quite broad and a number of lepidopteran cell lines are established (Lynn 2007), cell lines derived from *Spodoptera frugiperda* and *Trichoplusia ni* are mainly used in biotechnology. *Spodoptera frugiperda* *Sf*9 cells (Vaughn et al. 1977) are preferentially used for recombinant protein production as well as for baculovirus production. *Trichoplusia ni* BTI-TN-5B1-4 “High Five” cells (Granados et al. 1994; Wickham and Nemerow 1993) have been described to be more efficient in overall yield and especially in terms of secretion, suggested to be advantageous for complex and glycosylated proteins (Bruinzeel et al. 2002; Granados et al. 2007; Krammer et al. 2010). A new *Trichoplusia ni* cell line BTI-*Tnao*38 has been established (Hashimoto et al. 2010) and preliminary studies showed that this cell line is feasible for efficient protein secretion and indicated a higher stability during virus infection (Palmberger et al. 2012).

When generated by virus infection, recombinant proteins, their yield and quality, and the robustness of the production process are largely dependent on interactions of host and virus. Influential factors are cell line-specific parameters and genetic features of the virus. Characterization of these interconnections and their comparative analysis are required for setting up standardized, consistent processes that are eligible for generating products, fulfilling quality standards, production requirements and authorities’ regulations.

Here we present a comparative analysis of three different insect cell lines (*Sf*9, High Five, *Tnao*38) by analysing parameters influenced by host–virus interactions. As the cell lines in our study are of two different species’ origin, we sought to determine possible differences in order to expand cell line characterization and identify strategies for improvement of insect cell based production processes. The hypothesis regarding investigation of virus production in different cell lines was that virus produced in a certain cell line may exhibit an increased susceptibility if that virus was titered on that particular cell line. On the other hand, we wanted to validate other insect cell lines, e.g. *Tnao*38 cell line, in addition to *Sf*9 cells for baculovirus titration. Besides susceptibility and high titer virus production, we tested the chosen cell lines in terms of intra-cellular and secreted protein expression. The yellow fluorescent protein (YFP) and a secreted version of the influenza A virus hemagglutinin (HA) subtype H1 representing a complex, glycosylated, pharmaceutically relevant protein served as model proteins. This is the first systematic study that includes validation of insect cell lines for virus titer determination of viruses that have been generated in different cell lines, virus production yields as well as protein expression capacities.

## Materials and methods

### Cells and viruses

*Spodoptera frugiperda Sf9* cells (ATCC CRL-1711) (Vaughn et al. 1977), *Trichoplusia ni* BTI-TN-5B1-4 “High Five” (“Hi5”) cells (ATCC CRL-10859) (Granados et al. 1994; Wickham and Nemerow 1993) and *Trichoplusia ni* BTI-*Tnao*38 cells (Hashimoto et al. 2010) were maintained in custom modified, serum-free IPL-41 medium (PAN-Biotech) in T-flasks at 27 °C. IPl-41 medium was supplemented with 3 % (v/v) fetal calf serum (FCS) for growing *Sf*9 cells. Viral titers were determined by standard plaque assay using tenfold dilution series (*n* = 3).

### Construction of recombinant baculovirus

The Influenza Hemagglutinin H1 gene (A/California/04/09), as modified by Krammer et al. (2012), was PCR-amplified, the obtained product was digested with *Bam*HI/*Xba*I and ligated into p*Ac*Bac-1 vector (EMBL, Grenoble) that had been digested with the same enzymes, resulting in p*Ac*Bac-1-H1-Cal09. p*Ac*Bac-1-H1-Cal09 was further used for insertion into a MultiBac genome via Tn7 transposition in DH10MultiBacY cells according to Fitzgerald et al. (2006). Subsequently, recombinant baculovirus *Ac*NPV-H1-Cal09-YFP expressing soluble hemagglutinin and intracellular YFP was generated in *Sf*9 cells.

### Analysis of susceptibility of cell lines and baculovirus production

*Tnao*38, High Five and *Sf*9 cells were infected with a multiplicity of infection (MOI) of 1 with *Ac*NPV-H1-Cal09-YFP. Virus was harvested 6 days post-infection (dpi) and clarified by centrifugation at 1,000×*g* for 10 min. The obtained virus generated in each cell line was used for subsequent titer determination on all three cell lines via endpoint-dilution assay (Harwood 2007) in 96-well plates. Evaluation was done 6 dpi by fluorescence microscopy.

### Recombinant protein expression

9 × 10^5^ cells/ml of each cell line were infected with *Ac*NPV-H1-Cal09-YFP at an MOI of 10 in duplicates. Infected cells were maintained in shake-flasks in 50 ml at 100 rpm at 27 °C in HyClone SFM4Insect medium (Thermo Fisher Scientific) (*Tnao*38 (0 % (v/v) FCS) and *Sf*9 [3 % (v/v) FCS]) or serum-free IPL-41 medium (High Five). Cells from each cell line infected with virus encoding an unrelated protein served as negative control. Growth behaviour of infected cells was monitored, respective total and viable cell numbers were determined via TC20 cell counter (Bio-Rad). Infected cells were harvested every 24 h for 5 days and analyzed in regard to recombinant protein expression. Cell pellets were lysed in 1 ml I-PER Insect Cell Protein Extraction Reagent (Pierce) and YFP expression was measured in a plate reader at 488 nm excitation/520 nm emission. To assess HA expression, supernatant and pellet fractions were analyzed via SDS-PAGE and Western blot. Samples were mixed with 2× electrophoresis buffer containing 0.5 M Tris/HCl, 87 % (w/v) glycerol, 2 % (w/v) SDS, 0.1 % (v/v) Bromphenol Blue, pH 6.8 and 100 mM DTT. Proteins were separated by SDS-PAGE according to Laemmli, stained with Comassie Brilliant Blue G-250 or electroblotted on a PVDF transfer membrane. Membranes were blocked overnight with 3 % BSA in T-PBS (PBS with 0.1 % (v/v) Tween 20). Proteins were detected with an anti-H1-Cal09 polyclonal serum produced in mice, (EF-BIO, s.r.o., Tabaková 2942/5, 811 07, Bratislava, Slovakia) diluted 1:2,000 in T-PBS containing 1 % (w/v) BSA by incubation for 1 h. Followed by 1 h incubation of alkaline phosphatase conjugated anti-mouse IgG (γ-chain specific, produced in goat) antibody (Sigma-Aldrich, A1047) diluted 1:2000 in T-PBS containing 1 % (w/v) BSA, Western blots were developed using BCIP/NBT solutions (Sigma-Aldrich) (50 mg/ml).

## Results

### Virus–host interactions: analysis of susceptibility of cell lines and production of baculovirus progeny

**Fig. 1:**
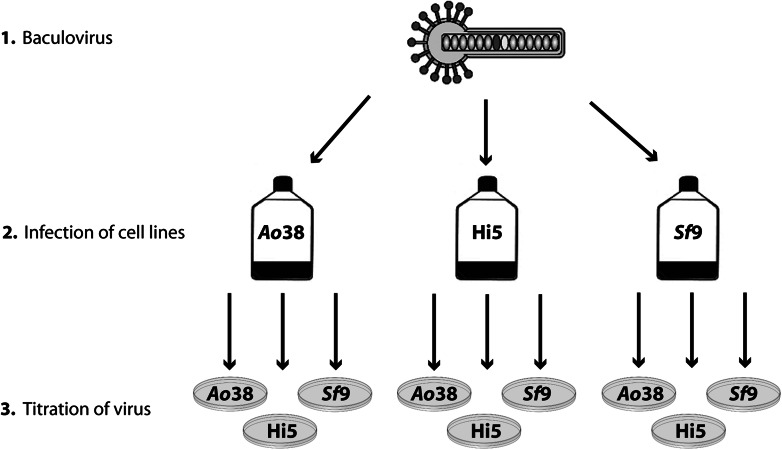
Schematic representation of baculovirus production and titer determination in and on *Tnao*38, High Five and *Sf*9 cells. *Tnao*38, High Five and *Sf*9 cells were infected with a YFP-encoding baculovirus. Virus generated in each cell line was subsequently titrated on all three cell lines. Titers were determined via endpoint dilution assay

Based on the observation that insect cell lines differ in susceptibility to *Ac*NPV (Lynn 2003), we wanted to investigate possible differences in virus up-take and titration results when in contrast to the routinely used *Sf*9 cells, High Five and *Tnao*38 cells are instead employed for titration. Therefore, all three cell lines were infected with *Ac*NPV-H1-Cal09-YFP generated in *Sf*9 cells at an MOI of 1. Respective titers were determined via endpoint dilution assay on all three cell lines in parallel (experimental scheme is shown in Fig. 1).

**Fig. 2:**
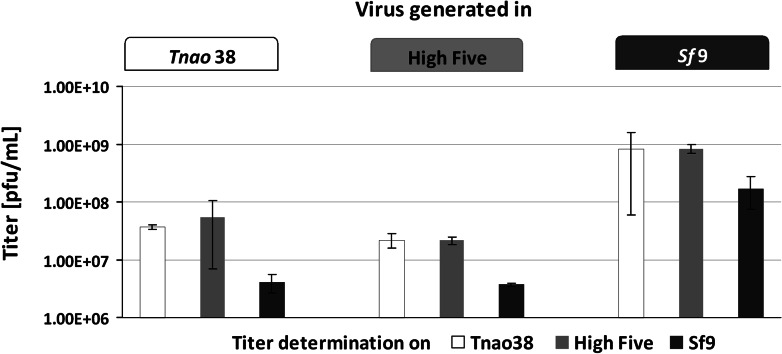
Analysis of susceptibility and baculovirus production in *Tnao*38, High Five and *Sf*9 cells. *Tnao*38, High Five and *Sf*9 cells were infected with *Ac*NPV-H1-Cal09-YFP at an MOI of 1, viral titers raised 6 days post infection were evaluated via endpoint dilution assay on all three cell lines. Titer values are given as plaque forming units (pfu)/ml

Figure 2 shows results for titer determination of virus generated by infection of the different cell lines on *Tnao*38, High Five and *Sf*9 cells.

Independent of the cell line employed for virus production, calculated titers were about one log higher when *Tnao*38 and High Five cells were used for titration as compared to *Sf*9 cells. Thus, both *Trichoplusia ni*-derived cell lines displayed higher susceptibility to *Ac*NPV than *Spodoptera frugiperda* *Sf*9 cells. Apparently, the producing host cell had no influence on viral up-take in the different cell lines. Furthermore, it was noted that *Sf*9 cells yielded about 100-fold more infectious particles as compared to *Tnao*38 and High Five cells, independent which cell line was used for titer determination. Thus, higher susceptibility is not combined with faster or more efficient virus replication or higher virus titer production.

### Recombinant protein expression

For validation of the three cell lines *Tnao*38, High Five and *Sf*9 in regard to recombinant protein expression, cells were infected at an MOI of 10 with *Ac*NPV-H1-Cal09-YFP. This baculovirus construct was designed to express YFP as an easy detectable protein that is produced intracellularly and a secreted version of influenza A virus hemagglutinin, lacking the transmembrane domain, as a model for a complex glycoprotein. Experiments were performed in duplicates, showing consistent results; representative data is shown for one replica.

#### Intracellular protein expression

**Fig. 3:**
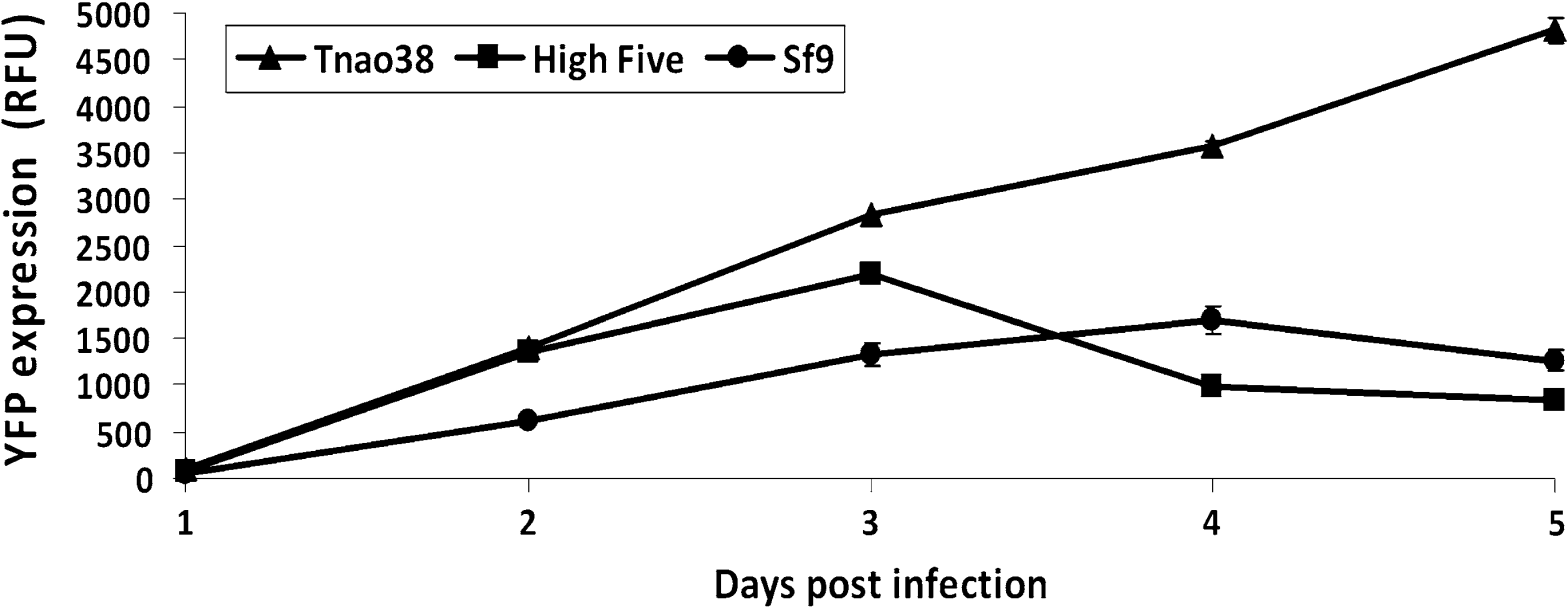
Intracellular YFP expression. *Tnao*38, High Five and *Sf*9 cells were infected with *Ac*NPV-H1-Cal09-YFP at an MOI of 10. Cells were harvested at indicated time post infection, cellular pellets were lysed and YFP expression was analyzed by fluorescence measurement in a plate reader. Values for relative fluorescence units (RFU) are given as the average from three technical replicas

Intracellular YFP yield was detected via fluorescence measurement in a plate reader (Fig. 3).

An increase of the YFP signal was detected in all cell lines 2 dpi. When comparing YFP peak levels, *Tnao*38 cells showed twofold higher expression than High Five cells and an about threefold higher YFP signal could be detected as compared to *Sf*9 cells. Overall, YFP expression in *Tnao*38, High Five and *Sf*9 cells showed cell line dependent differences, in terms of yield and also regarding time point of production peaks. Additionally cell viability was determined over time for all three cell lines, where *Tnao*38 cells showed the highest value on day 5 (60 %) in comparison to High Five (16 %) and *Sf*9 cells (32 %). This may indicate a higher stability of the *Tnao*38 cell line which has been described previously (Palmberger et al. 2012), suggesting that this may be the cause for higher YFP expression.

#### Protein secretion

Protein yields and integrity of soluble trimeric influenza A virus hemagglutinin H1-Cal09 were detected by Western blot analysis.

**Fig. 4:**
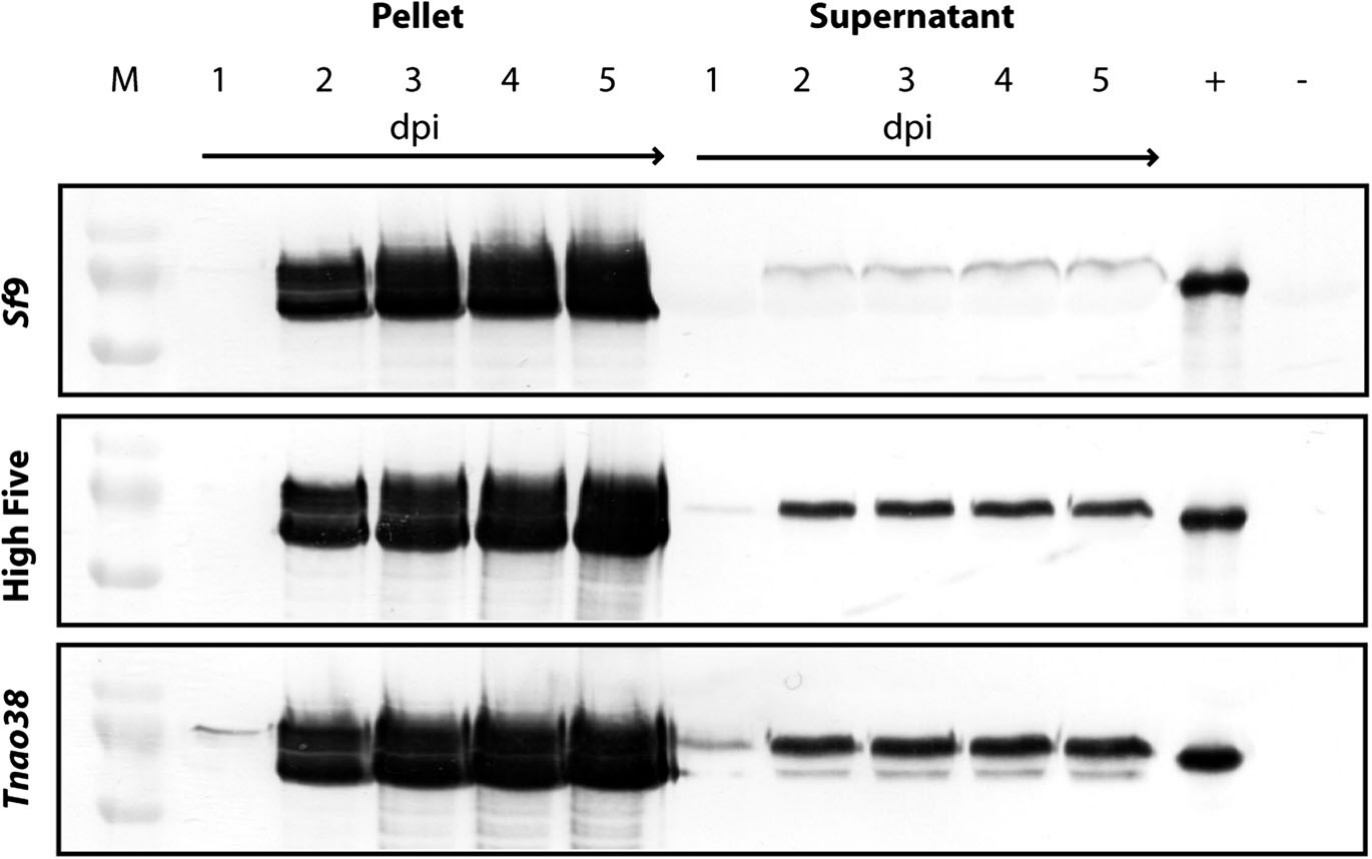
Hemagglutinin expression in *Tnao*38, High Five and *Sf*9 cells. *Tnao*38, High Five and *Sf*9 cells were infected with *Ac*NPV-H1-Cal09-YFP at an MOI of 10. Cells were harvested at indicated time post infection and pellet and supernatant fractions were subjected to Western blot analysis with mouse anti-H1-Cal09 polyclonal serum. *M* protein standard, *1–5* Pellet/Supernatant fractions at indicated days post infection (dpi), *+* (*positive control*): Ni–NTA purified HA (H1-Cal09), *−* (*negative control*) unrelated protein

Figure 4 shows unsecreted and secreted HA in the cell pellet and in the supernatant of *Sf*9, High Five and *Tnao*38 cells, respectively. Strong expression of HA was detected 2–5 days after infection in the cell pellet, for all cell lines. Secreted HA was detected in the supernatant of all cells starting on day 2 throughout to day 5, where significantly higher amounts were found for High Five and *Tnao*38 cells as compared to S*f*9 cells. Yet, some product degradation was observed for secreted HA from *Tnao*38 cells (Fig. 4, lower panel), while in the supernatant of High Five cells, only one clear band was visible, which corresponds to the size of the fully processed HA. In contrast, all cell pellets resulted in a double band, probably due to uncompleted processing or partial degradation.

## Discussion

Production of biopharmaceuticals in insect cells is an emerging field in biotechnology. Cell engineering and vector design for higher expression rates and for providing human-like glycosylation are successful strategies for further advancement of the baculovirus expression system in terms of protein quality (Aumiller et al. 2012; Viswanathan et al. 2005). When it comes to process design and up-scaling strategies, the potential and limits still need to be determined. One major factor is the choice of the insect cell line. While *Sf*9 cells are the most widely used cell line, High Five cells have shown to be highly feasible for efficient protein secretion (Granados et al. 2007; Krammer et al. 2010). *Tnao*38 is a recently established cell line (Hashimoto et al. 2010) and only preliminary data about production performance have been published. Therefore, comparative analysis and validation of available insect cell lines is highly important.

Here, we characterized these three cell lines, *Tnao*38, High Five and *Sf*9, in terms of baculovirus susceptibility, virus progeny production and in regards to their feasibility to express non-secreted and secreted proteins. Employment of *Tnao*38 and High Five cells for baculovirus titer determination resulted in a tenfold higher titer as compared to using *Sf*9 cells, independently which cell line was used for virus propagation (Fig. 2). Thus, employment of *Tnao*38 and High Five cells provides a more sensitive assay for titer determination, which can be of importance e.g. for the detection of unwanted baculovirus background and for fulfilling regulatory requirements for licensing new baculoviral derived products of medical relevance. According to our findings, *Sf*9 cells as state of the art cell line used for determination of the baculovirus titer does not reflect the actual number of infectious virus particles, but leads to an underestimation of titer values.

Correct titer results are important for calculating MOIs; infection with more virus than anticipated may increase metabolic burden and cause decreased productivity. Since many processes have been optimized regarding MOI for transduction experiments in mammalian cell culture and gene therapeutic applications, the fact that the titer is about one log higher than estimated according to *Sf*9 cell based titer determination might be taken under consideration. Possibly, there exist insect cell lines that would give an even higher titer value and our findings may just reflect an approximation of the ‘real’ baculovirus titer. Also, accurate titer determination based on more susceptible cells may help in the establishment of a baculovirus reference material for inter- and intra-laboratory comparison (Kamen et al. 2011). *Sf*9 cells are preferably used for basic viral assays, transfections and for the production of high titer baculovirus stocks. In terms of producing baculoviruses, we found *Sf*9 cells to be 100-fold more efficient. Regarding recombinant protein expression, we found that *Tnao*38 and High Five cells were more suitable for both intra- and extracellular model proteins. *Tnao*38 cells showed approximately a 2- and 3-fold higher intracellular YFP expression compared to High Five and *Sf*9 cells, respectively (Fig. 3). The persistent increase of intracellularly-expressed YFP in *Tnao*38 cells may indicate that this cell line exhibits higher stability upon baculovirus infection.

With regard to expression of the influenza A virus hemagglutinin, *Tnao*38 and High Five cells were comparable in terms of secretion and overall expression. In contrast to YFP expression experiments, here we found similar expression levels on days 2–5. This might be due to the higher stability of the trimeric HA as compared to YFP. Furthermore, no difference in the course of the expression was observed, suggesting similar properties in robustness. In terms of product quality, High Five cells seem to be superior as compared to *Tnao*38 cells, since Western blot analysis indicated protein degradation in the supernatant of *Tnao*38 cells, while HA secreted by High Five cells resulted in only one distinct band. *Sf*9 cells showed significantly lower amounts of secreted HA in the supernatant as compared to *Tnao*38 and High Five cells (Fig. 4). Higher protein yields may be attributed to lower metabolic burden due to virus replication and to the higher susceptibility of the two *Trichoplusia ni* cell lines. To date, still many secreted glycoproteins are generated in *Sf*9 cells, such as influenza virus HA and various virus-like particles (Cox 2008; Khurana et al. 2011; Liu et al. 2013; Tretyakova et al. 2013). However, our findings suggest the high applicability of cell lines derived from *Trichoplusia ni* for recombinant protein expression, especially in the case of secreted proteins. Further investigation of virus-host interactions and their influence on productivity, as well as tools for reliable quality control of the products, will be necessary to advance the application of insect cells as feasible cell factories.
